# Anterior Drawer Maneuver to Access Knee Posteromedial Compartment

**DOI:** 10.1002/atn2.70076

**Published:** 2026-07-13

**Authors:** Sheetal Gupta, Gaurav Jindal, Parvind Singh, Abhishek Kulkarni, Sandeep Tukade

**Affiliations:** ^1^ Galaxy Hospital Bhopal Madhya Pradesh India

## Abstract

Over the years, diagnostic arthroscopy has been the key to accurately diagnosing lesions in the knee. However, accessing the posterior compartment of the knee remains a challenge for upcoming arthroscopy surgeons. Gillquist was the first surgeon to publish a technique for accessing the posteromedial compartment in 1979, following which several techniques were developed, like modified Gillquist and switching stick maneuvers. According to Gillquist, “posterior compartments are the litterbox of the knee” and the advantages of posterior evaluation include evaluation of ramp lesions, remnants after partial meniscectomy, loose bodies, and to safely create posterior portals for arthroscopic visualization, posterior cruciate ligament (PCL) surgery and ramp repair. However, there is a risk of damaging the lateral portion of the medial femoral condyle chondral damage and broken arthroscope while attempting these blind maneuvers, and these techniques require expertise and more operative time. Our approach aims to simplify these procedures, reducing the time require to access the posteromedial compartment while minimizing the risk of unintended damage to the medial femoral condyle cartilage.

## Graphical abstract

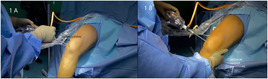

**VIDEO 1:** Anterior drawer maneuver to access knee posterior‐medial compartment. No disclosures. Hello, this is my presentation of a right knee anterior cruciate ligament tear where I'm showing with the help of a probe of a triangle which is formed by lateral surface of medial femoral condyle and the posterior cruciate ligament and the anterior cruciate ligament. As we advance our scope towards the triangle and give anterior door pull with the help of opposite hand, a space is created and we push our scope into this space. As soon as we leave the anterior drawer, we enter the posterior middle compartment. We can do the needle test to check the pathology. In a case two of left knee ACL tear, viewing from anterior lateral portal, I advance my scope towards the triangle and give an anterior drawer pull from the opposite hand. Then, I push my scope towards the space created and leaves the anterior drawer pull, and I enter into the posterior medial compartment. This is outside view of performing a technique of anterior drawer with leg in hanging position. Now, we examine the medial compartment in meniscus with the help of a probe test we found to be normal, but as we enter the posterior medial compartment, we found a large ramp tear. Similarly, in another case, we found meniscus to be normal, but as we enter the posterior medial compartment, we found a large ramp tear confirmed by a needle test. Then, we made two posterior middle portals high and low and view the ramp tear from the high posterior middle portal and probe it from lower one. Then, we repair this ramp tear with the help of a suture lasso technique very easily. In another case, we found middle meniscus to be normal confirmed by a probe test. But as we enter the posterior medial compartment, we found a large ramp tear. Then, we made two posterior medial portals and diamond rasped the lesion (pathology) and shifted to the posterior medial side. While viewing from the high posterior medial portal, we repair the ramp tear with the help of a knee scorpion from the lower posterior medial portal. As we pass the suture from the meniscal side and the capsular side, we shifted these sutures with the help of a suture retriever from this very triangle as we used for the anterior draw maneuver technique. Then, we again pass another suture from the meniscus and capsular layer and shifted again these sutures to the anterior side with the help of a suture retriever. Then, we tie the knot from lower posterior middle portal and confirm it with the help of a probe test. Thank you for watching the video. Video content can be viewed at https://doi.org/10.1002/atn2.70076.

The technique for accessing the knee joint posterior compartment was first published by J. Gillquist^1^ in 1979. His initial method involved inserting a camera into the transpatellar tendon portal, which is a portal located 1 cm below the patellar tendon apex. A 5 mm Karl‐Storz, 30° arthroscope was inserted via the portal and maintained between the laterally facing medial femoral condyle (MFC) and the posterior cruciate ligament (PCL) to access the posterior compartment. After that, a 70° scope is used in place of a 30° arthroscope to provide a wider field of vision. As a result, the meniscus, its posterior horn, the meniscocapsular junction, PCL, and loose bodies in the posterior compartment can all be seen.

In a study by McGinnis et al, a 5 mm bur (ConMed, Germany) was inserted through the anteromedial portal and used to perform a limited inferior notchplasty at the lateral aspect of the MFC until the posterior horn of the medial meniscus was visualized. A 30° arthroscope was then advanced until the posteromedial compartment of the knee could be observed. Then, the arthroscopic sheath was left in place, and a 70° arthroscope was introduced.^2^

In later studies by Lubowitz et al.^3^ and Kramer et al.,^4^ the posteromedial compartment was accessed indirectly via the blunt obturator technique instead of direct arthroscope introduction.

Difficulties encountered while inserting the arthroscope to visualize the posteromedial compartment include 1) Narrow intercondylar notch: Inferior notchplasty of the lateral aspect of the MFC is done to widen the intercondylar notch and ease the passage of the scope through the interval between PCL and MFC.^2^ 2) Blind passage of the scope in the knee can inadvertently damage the meniscus, cartilage, and the camera scope. 3) These techniques have been reported to cause mild damage in 22% of the knees, severe damage in 3% of the knees, and failure to enter the posterior compartment of the knee in 3% of cases.^5^ We describe a technique for accessing the posteromedial compartment of the knee in anterior cruciate ligament (ACL)‐deficient patients with minimum operative steps and avoiding any inadvertent damage to the intra‐articular structures.

## SURGICAL TECHNIQUE

Patients with ACL injury with or without concomitant meniscus (including bucket handle tears), meniscocapsular, and collateral ligament injuries are identified. Patients with isolated PCL, meniscus, and extraarticular ligament injuries are excluded. A thorough clinical evaluation via history and physical examination should be done; then, radiographic and magnetic resonance imaging performed to diagnose the pathology. Informed and written consent was obtained from the patients regarding the following technique, and patient privacy was maintained.

**FIGURE 1:**
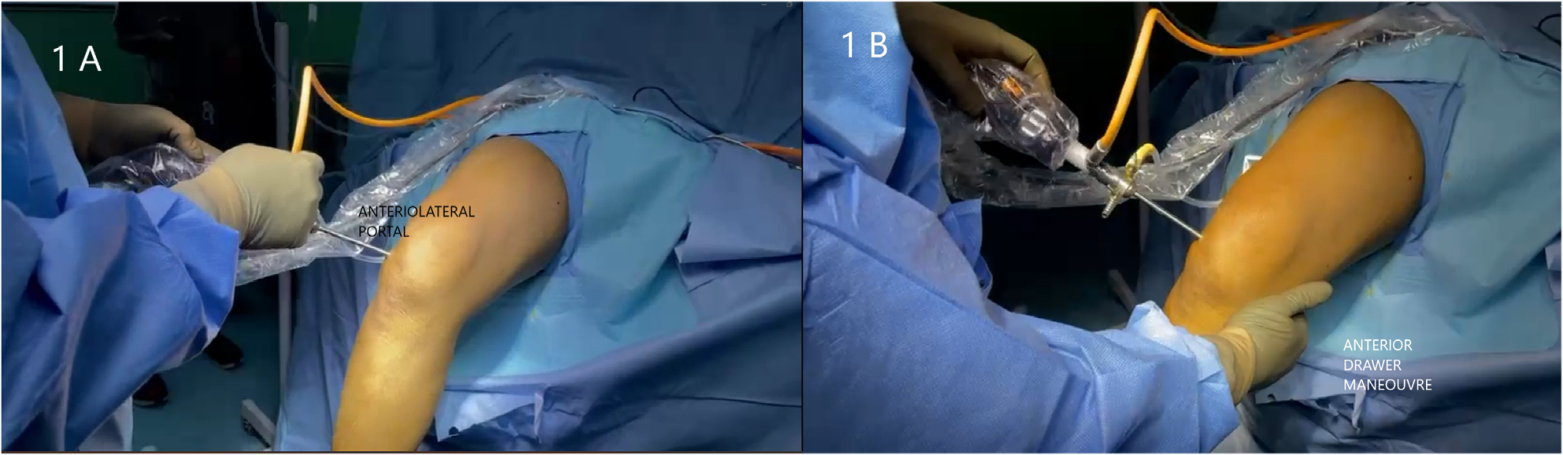
(A) Right knee with the leg hanging in a holder position; arthroscope introduced through the Al portal. (B) Arthroscope in the Al portal showing the anterior drawer maneuver to access the PM compartment. (Al, anterolateral, PM, posteromedial.)

Knee should be in 90° of flexion, with preferably torn ACL, as prerequisites for the surgical technique. A supine position with the knee hanging, with a bolster below the affected knee, and the normal hip and knee in an abducted position is used (Figure 1).

Anterolateral portal was used as a viewing portal, and sometimes transpatellar portal was used to clear the fat pad if required (Video 1, Figure 1A).

### Steps of the Technique

**FIGURE 2:**
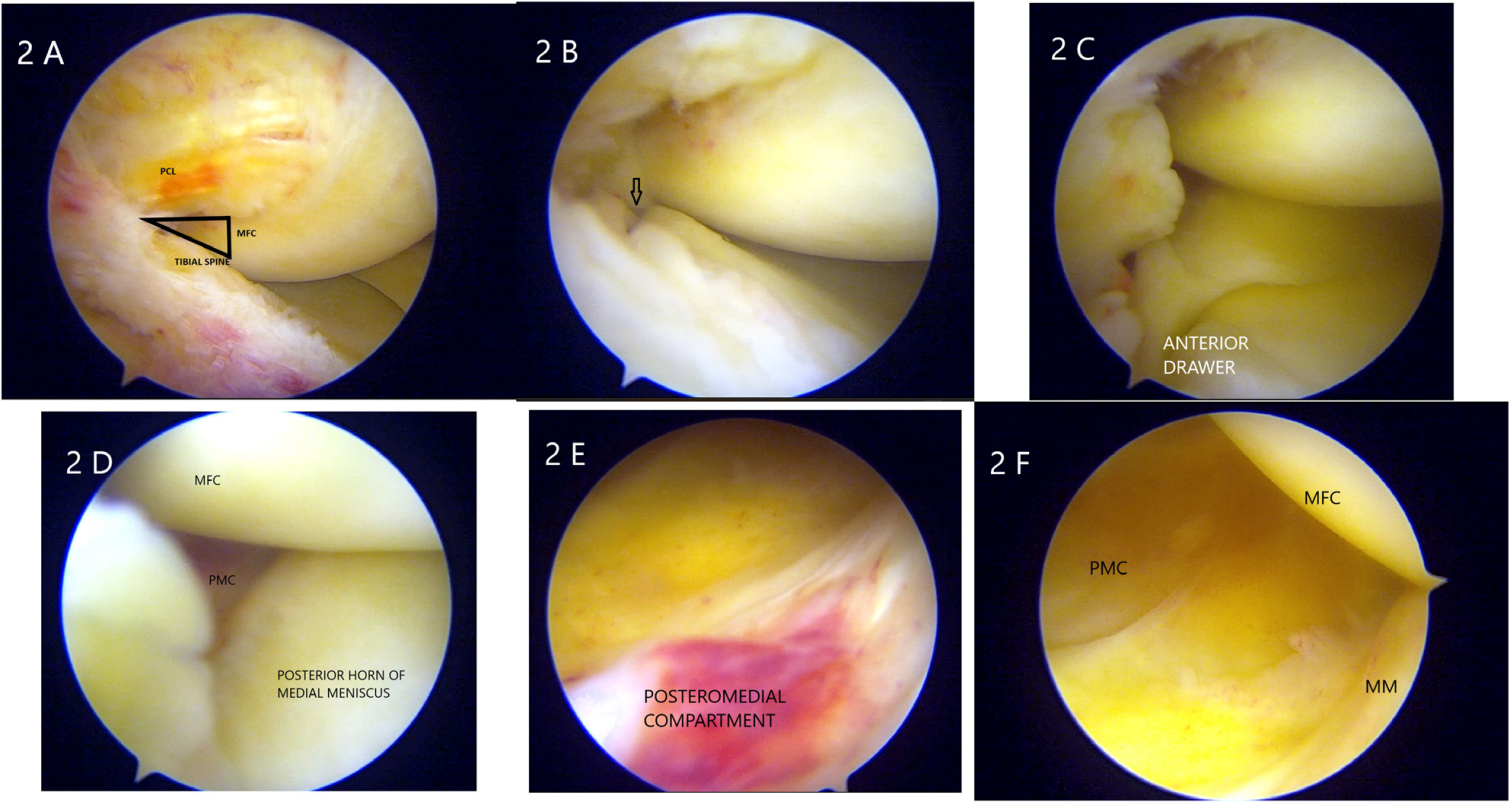
(A) Right knee, knee in 90° flexion, visualization from Al portal, showing a triangle of three structures with adjacent formed by PCL, the opposite formed by the lateral surface of MFC, and the hypotenuse formed by the remnant of ACL and the tibial spine. (B) Right knee, knee in 90° flexion, visualization from Al portal; as we introduced the scope in between the margins of the triangle as shown in Figure 2a, a small space starts to visualize behind the posterior border of medial meniscus root. (C) Right knee, knee in 90° of flexion, visualization from Al portal; performing anterior drawer pull, the space behind the posterior border of mm root enlarges. (D) Right knee, knee in 90° of flexion, visualization from Al portal; push our scope towards that enlarged space under direct visualization. (E) Right knee, knee in 90° flexion; while performing anterior drawer maneuver, the scope enters posteromedial compartment, and the anterior drawer pull stops. (F) Right knee, knee in 90° flexion; showing posteromedial compartment.

1. The anterolateral portal is placed 1 cm above the joint line and just next to the patellar tendon in a palpable soft spot.^6^ Begin by introducing the 30° arthroscope in 90° of knee flexion via the anterolateral portal (Figure 2).
2. Via the transpatellar tendon portal, introduce the shaver to clear the ligamentum mucosum and transpatellar fat pad in the intercondylar notch, if necessary.
3. Place the arthroscope between the PCL and lateral aspect of MFC (Figure 2B).
4. Give an anterior drawer pull on the tibia and push the scope under visualization (Figure 1B).
5. As soon as you leave the anterior drawer, the scope enters the posteromedial compartment (Figure 2F).
6. Posteromedial structures such as PCL, posterior horn of the medial meniscus, meniscocapsular structures, loose bodies, and synovial pathology in the posteromedial compartment could be assessed and managed.

## DISCUSSION

This is a safe, inexpensive, and easy‐to‐learn technique to enter the posteromedial compartment of the knee while performing arthroscopy. There is no risk of damaging the chondral structures and surrounding soft tissue structures. However, the only drawback to this technique is that it is difficult to perform in ACL‐intact knees (Table 1).

**TABLE 1:** Advantages and Disadvantages of Arthroscopic Anterior Drawer Technique to Access Posteromedial Knee Compartment

Advantages	Disadvantages
a. Ease of passage	a. Difficult to perform in the presence of intact anterior cruciate ligament
b. No switching‐stick portal required	
c. Damage to articular cartilage: less	
d. Time required to enter the posteromedial compartment: 4 to 5 seconds	

The original Gillquist maneuver,^1^ as described, has been reported to have an incidence of broken cameras and damaged equipment. Rather than using the transpatellar tendon portal for transcondylar posterior compartment vision, the modified Gillquist maneuver uses the anterolateral and anteromedial portals for arthroscope introduction.

To avoid damage to intra‐articular structures, Lubowitz et al.^3^ described a technique where they replaced the blind insertion of the arthroscope with the blind insertion of the obturator through the arthroscopic cannula. Kramer et al.^4^ similarly described this technique, where they introduced the blunt obturator through the anterolateral portal.

As these techniques required blind insertion of an obturator rod or trocar cannula, multiple attempts were still required to access the posteromedial compartment, and the risk of damaging intra‐articular structures was high.

Lee et al. described a technique of a switching stick to enter the posteromedial compartment of the knee. They guided the switching stick to the posteromedial compartment via the anterolateral portal with the camera in the anteromedial compartment. This lowers the risk of damage to the intra‐articular structures since the entry into the posteromedial compartment is under vision, but this technique requires more operative time.^5^

Multiple techniques have been described to enter the posteromedial compartment of the knee without damaging the intra‐articular structures. In comparison to other techniques, such as the switching stick, which require additional steps and the use of 30° and 70° arthroscopes, this technique is fast, easy to perform, and does not require any extra steps. Hence, this method can be used safely to access the posteromedial compartment even by beginner surgeons with a more predictable outcome.

## DISCLOSURES

The authors (S.G., G.J., P.S., A.K., S.T.) declares the following financial interests/personal relationships which may be considered as potential competing interests: S.G., G.J., P.S., A.K., S.T. report an employment relationship with Galaxy Hospital, Bhopal.
